# Three new species of *Heteromastus* (Annelida, Capitellidae) from Korean waters, with genetic evidence based on two gene markers

**DOI:** 10.3897/zookeys.869.34380

**Published:** 2019-08-05

**Authors:** Man-Ki Jeong, Ho Young Soh, Hae-Lip Suh

**Affiliations:** 1 Fishery resource management research center based on ICT (FMRC), Chonnam National University, Daehak-ro, Yeosu 59626, South Korea; 2 Faculty of Marine Technology, Chonnam National University, Daehak-ro, Yeosu 59626, South Korea; 3 Department of Oceanography, Chonnam National University, Yongbong-ro, Buk-gu, Gwangju 61186, South Korea

**Keywords:** Genetic comparison, histone H3, morphology, mtCOI, new species

## Abstract

Three undescribed species of *Heteromastus* Eisig, 1887 were collected from intertidal to sublittoral habitats in western and southern waters of Korea. *Heteromastus namhaensis* sp. nov. is distinguishable from other congeners by the presence of hemispheric notopodial lobes in the posterior abdomen. *Heteromastus gusipoensis* sp. nov. closely resembles *H. tohbaiensis* Yabe & Mawatari, 1998 in the absence of posteriorly extended abdominal notopodial lobes, but differs in the absence of eyespots on the prostomium and distinct node on the shaft of thoracic hooks in *H. gusipoensis*. *Heteromastus koreanus* sp. nov. is similar to *H. filiformis* sensu Hutchings & Rainer, 1982 in the shape of abdominal notopodia, but clearly differs in dentition of the abdominal hooks and methylene green staining pattern (MGSP). DNA sequences (mtCOI and histone H3) of these new Korean species were compared with all sequences of *Heteromastus* species available in the public database. Molecular results showed distinct genetic differences among these three new Korean species at species level. Comparison of mtCOI gene revealed significant genetic difference between *H. filiformis* and these Korean species. A comprehensive comparison between three *Heteromastus* species of present study and their closely related congeners is conducted based on morphological and genetic results.

## Introduction

The genus *Heteromastus* Eisig, 1887, which belongs to the family Capitellidae Grube, 1862, is commonly found from intertidal areas to shallow subtidal depths in a variety of sediment types, including fine and silty sand and mud (Blake 2000; Dean 2001). Feeding activity of *Heteromastus* plays an important role in the supply of overlying oxygenated water into anoxic muds below the redox potential discontinuity (Cadée 1979). *Heteromastus* is known as a biological indicator and opportunistic species in marine hypoxia condition (Cadée 1979). The genus *Heteromastus* was first designated by Eisig (1887) based on the description of *H. filiformis* Claparède, 1864 (as *Capitella filiformis*) from southern France. According to his diagnosis, *Heteromastus* is distinguished from other genera in the family by having 11 thoracic chaetigers, of which the first five only have capillaries. Green (2002) improved this generic definition by including the differences in the thoracic (long-shafted) and abdominal (short-shafted) hooks. However, the lack of good generic characteristics has led to taxonomic confusion in this genus (Blake 2000; Green 2002). For instance, although *Heteromastus* currently contains seven valid species (Read and Fauchald 2018), the chaetal arrangement of *H. giganteus* Zachs, 1933 does not match to the original generic definition (Zachs 1933). Among the recognized generic characteristics, the number of thoracic segments can be miscounted due to ambiguous boundaries among peristomium, thorax, and abdomen (Blake 2000). In addition, the thoracic chaetal arrangement varies depending on the degree of development (Fredette 1982).

*Heteromastus filiformis* (Claparède, 1864), the generic type species, is well known as a cosmopolitan species found in various types of the habitats and has been referred to in many ecological studies (Hutchings and Rainer 1982). Species-specific characters of this representative species have been controversial due to incomplete original description and the absence of the original type specimens from southern France, although Hutchings and Rainer (1982) have later designated the neotype from Egypt (Green 2002). In addition, the dental structure of abdominal hooks and the shape of posterior parapodial lobes of *H. filiformis* have been described differently in published records including the neotype (Blake 2000; Green 2002). In Korean waters, Choi and Yoon (2016) have reported that *H. filiformis* was the only species belonging to genus *Heteromastus* occurring in this region based on morphological features. They have suggested that Korean specimens have some minor differences with former records of *H. filiformis* in the morphology of abdominal hooks and methylene green staining pattern, although these characters have been used for identification of recorded species in family Capitellidae (Blake 2000; Green 2002; Jeong et al. 2017b). Recently, a combination of morphological and molecular analysis has been conducted to distinguish very close polychaete species and geographical populations (e.g. Glasby et al. 2013; Jeong et al. 2017b, 2018). The aim of the present study is to verify the taxonomic status of undescribed *Heteromastus* species inhabiting Korean waters based on morphological and molecular analysis using two different partial genes (mtCOI and histone H3) in comparison with their closest species in the genus.

## Materials and methods

### Morphological analysis

**Figure 1.**
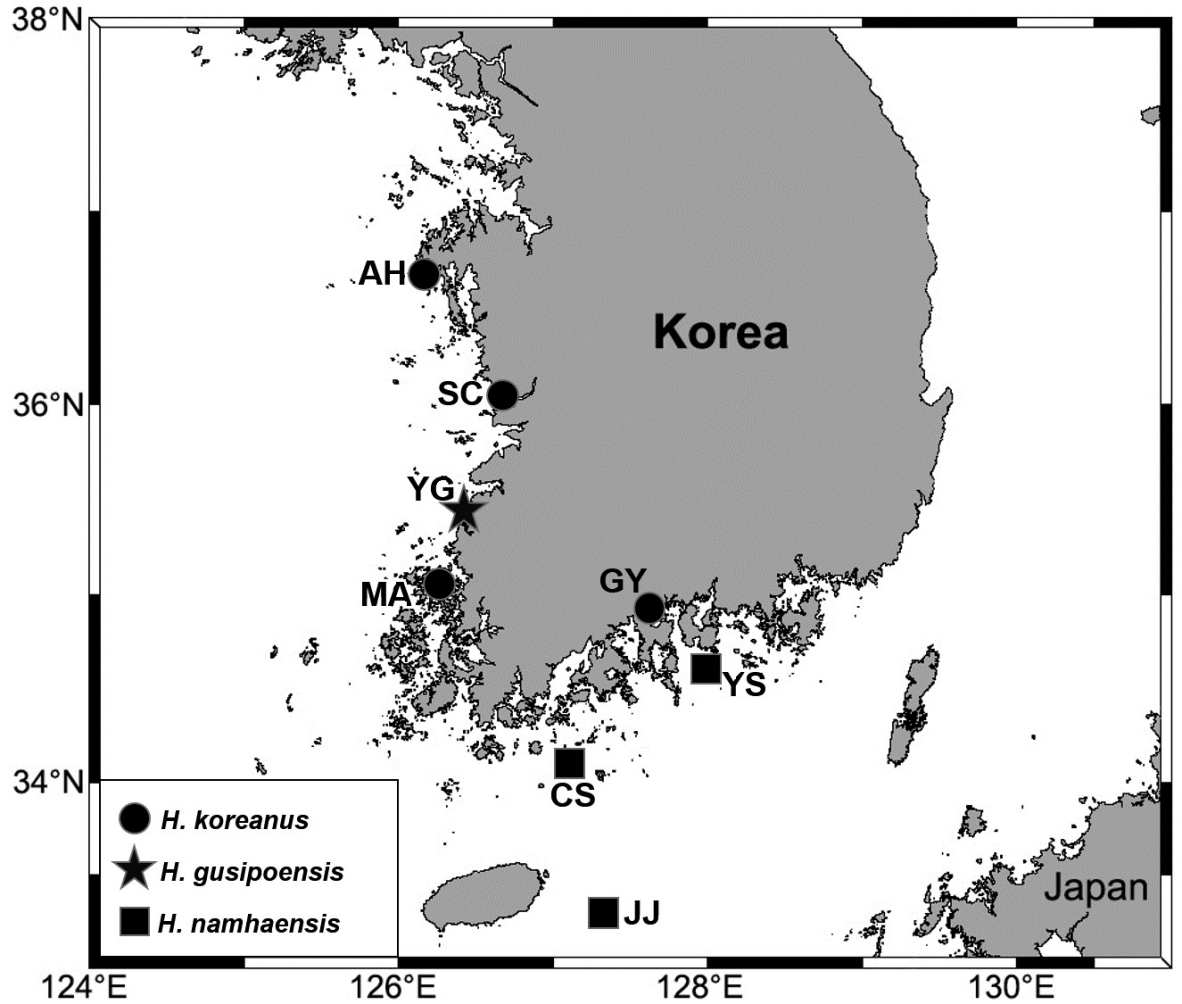
Map of study area with main collecting locations indicated. Abbreviations (district names): AH, Anheung; CS, Cheongsando; GY, Gwangyang; JJ, Jejudo; MA, Muan; SC, Seochun; YG, Yeonggwang; YS, Yeosu.

Samples were collected from eight stations of Korean sublittoral areas using a 0.05 m^2^ Van Veen grab (Fig. 1). Sediment samples were elutriated over a 0.5 mm sieve in a 30 L seawater container and organisms were transferred to a 1 L collecting jar containing 7% MgCl_2_ solution for anesthesia. Relaxed samples were fixed in a buffered solution of 10% formalin within one hour and then finally preserved in 95% ethanol. In the laboratory, *Heteromastus* specimens were sorted under a Zoom Stereomicroscope (SMZ745T, Nikon). Line drawings were performed using a differential interference contrast microscope (Eclipse Ci-L, Nikon) and a digital pen display (Cintiq 22HD, Wacom). Methyl green staining patterns (MGSP) and scanning electron microscopy (SEM) analyses were performed as delineated by Jeong et al. (2017b). The examined type materials were deposited in the collection of Marine Biodiversity Institute of Korea (MABIK) in Seocheon, Korea (Table 1).

**Table 1.** A list of sampling localities, species name, sample type, voucher number, Genbank accession number, and references. AC: Accession number, BOLD: Barcode of life data system (http://www.boldsystems.org).

**Species name**	**Location**		**Latitude / Longitude (DDM)**	**Type**	**Voucher number**	**Accession number of Genbank**		**References**
	**Country**	**District**				**mtCOI**	**Histone H3**	
*H. namhaensis* sp. nov.	Korea	Cheongsando	34°1.662'N, 127°4.272'E	Holotype	NA00155558	MK032276	MK032285	This study
		Jejudo	33°16.699'N, 127°16.230’ E	Paratype	NA00155559	MK032277	MK032286	
		Yeosu	34°41.569'N, 127°51.848'E	Paratype	NA00155560	MK032278	MK032287	
*H. gusipoensis* sp. nov.		Yeonggwang	35°25.819'N, 126°25.482'E	Holotype	NA00155561			
				Paratype	NA00155562	MK032279	MK032288	
				Paratype	NA00155563	MK032280	MK032289	
				Non-type	NA00155564	MK032281	MK032290	
*H. koreanus* sp. nov.		Muan	35°6.270'N, 126°20.093'E	Holotype	NA00155565			
		Anheung	36°40.740'N, 126°9.121'E	Paratype	NA00155566	MK032282	MK032291	
		Gwangyang	34°55.940'N, 127°36.252'E	Paratype	NA00155567	MK032283	MK032292	
		Seochun	36°0.95'N, 126°39.79'E	Non-type	NA00155568	MK032284	MK032293	
*H. filiformis*	China	Bohai Sea	38°N, 120°E		BIOUG03550-H04	HZPLY183-12 (AC of BOLD)		BOLD (2019)
*H. filiformis*	USA	Maryland	38°52.428'N, 76°31.482'W		USNM:IZ:1463490	MH235890		Unpublished

### Molecular analysis

Genomic DNA was extracted from ethanol-preserved specimens. Specimens used for molecular analysis were partially dissected (ca 2 segments) in the middle part of the abdomen. To extract genomic DNA, 1.5 mL centrifuge tubes each containing 45 μL of 10% Chelex suspension (Bio-Rad Laboratories Inc.), 5 μL of Proteinase K (10 mg/ml, iNtRON Biotechnology, Inc.), and dissected tissues (ca 2 segments) were incubated at 56 ℃ for 3–12 hours. Extracted genomic DNA was used as a template to amplify the target region. Polymerase chain reaction (PCR) was performed on a MasterCycler PCR thermal cycler (Eppendorf Co.). The primer pair for mtCOI was LCO1490 and HCO2198 (Folmer et al. 1994). For histone H3, it was H3F and H3R (Colgan et al. 1998). PCR mixtures contained 17 μL of deionized water, 1 μL of each primer (10 μM), 1 μL of DNA template and PCR premix (20 μL, BiONEER Co.). The temperature profile was as follows: 94 °C/180 s–(94 °C/30 s–46 °C/30 s–72 °C/60 s) * 40 cycles–72 °C/480 s for mtCOI and 94 °C/180 s–(94 °C/45 s–50 °C/60 s–72 °C/60 s) * 35 cycles–72 °C/420 s for histone H3. Purification and sequencing of obtained PCR products were performed at Macrogen Inc. facilities (Seoul, Korea). Forward and reverse sequences were edited using Chromas software version 2.3 (Technelysium Pty Ltd). Partial sequences of the mtCOI and histone H3 genes were aligned with the available sequences obtained from GenBank (http://www.ncbi.nlm.nih.gov/Genbank) and BOLDSYSTEMS (http://www.boldsystems.org/) using the Molecular Evolutionary Genetics Analysis (MEGA) software version 7.0 (Kumar et al. 2016). Table 1 summarizes information for all sequences used in the analyses. These aligned sequences were used as data sets to generate genetic distance using Kimura’s two-parameter (K2P) model (Kimura 1980). Based on K2P distances, intraspecific genetic differences within the Korean specimens and the interspecific genetic differences among the closest taxa were calculated.

## Results

### Systematics

#### Family Capitellidae Grube, 1862

##### Genus Heteromastus

Taxon classificationAnimaliaAnnelidaCapitellidae

Eisig, 1887

61678E48FBB55693B93CCA0AE5CF01AB

###### Type species.

*Heteromastus filiformis* (Claparède, 1864).

###### Type locality.

Port-Vendres, France.

###### Generic diagnosis

(modified after Magalhães and Blake (in press)). Prostomium short to long, conical, eyespots present or absent. Thorax with 11 chaetigers. Chaetiger 1 biramous. Chaetigers 1–5 with only capillary chaetae, chaetigers 6–11 with long-shafted hooded hooks. Abdominal chaetigers with short-shafted hooded hooks. Branchiae present or absent on posterior abdomen. Genital pores presence on posterior thoracic chaetigers. Lateral organs distinct on thorax and indistinct on abdomen. Pygidium adorned with ventral caudal cirrus.

##### Heteromastus namhaensis sp. nov.

Taxon classificationAnimaliaAnnelidaCapitellidae

C9B63C59DB6A57749F75A5A34E5E384C

http://zoobank.org/D41E7B49-B712-42A7-8095-94E906ABB121

**Figure 2.**
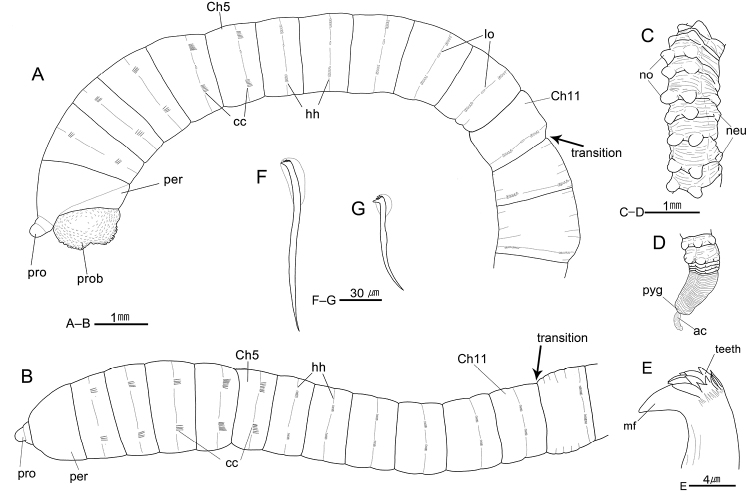
*Heteromastus namhaensis* sp. nov. **A** anterior end, left lateral view (holotype, NA00155558) **B** same, dorsal view **C** posterior abdominal segments, right lateral view (holotype, NA00155558) **D** posterior end, dorsal view (holotype, NA00155558) **E** abdominal short-shafted hook, frontal view. **F** thoracic long-shafted hook, lateral view **G** abdominal short-shafted hook, lateral view. Abbreviations: ac, anal cirrus; cc, capillary chaetae; Ch, chaetiger; gp, genital pore; hh, hooded hooks; lo, lateral organ; mf, main fang; neu, neuropod; no, notopod; per, peristomium; pro, prostomium; prob, proboscis; pyg, pygidium.

Figures 2A–G , 5A, B , 6A

###### Material examined.

**Holotype**: MABIKNA00155558, sex uncertain, Cheongsando, 34°1.662'N, 127°4.272'E, subtidal, sandy mud bottom, 34 m depth, March 2016, coll. Man-Ki Jeong. **Paratypes** (two specimens): MABIKNA00155560, Yeosu, 34°41.569'N, 127°51.848'E, subtidal, sandy mud bottom, 15 m depth, June 2018; MABIK NA00155559, Jejudo, 33°16.699'N, 127°16.230'E, subtidal, sandy mud bottom, 54 m depth, April 2018. Additional 6 specimens from type locality on SEM stub.

###### Diagnosis.

Abdominal hooks with four rows of teeth, three teeth in basal row, three in second and third row, and four to six in superior row. Genital pores present in intersegmental furrows between chaetigers 7–8, 8–9, 9–10, and 10–11. Hemispheric notopodial lobes present on posterior abdominal segments.

###### Description.

Holotype entire, about 60 mm long, 0.9 mm wide for 98 chaetigers (terminal part missing). Paratypes range from 19–41 mm in length, 0.5–0.8 mm width for 41–95 chaetigers. Body thread-like, rounded dorsally, flattened ventrally, widest in anterior thoracic chaetigers, and tapering from abdomen to pygidium. Color brownish yellow in alcohol.

Prostomium conical, with short and hemispherical palpode; nuchal organs not seen, eyespots absent (Fig. 2A, B). Everted proboscis with numerous small papillae (Fig. 2A). Peristomium uni-annulated and slightly longer than first thoracic chaetiger (Fig. 2A, B).

Thorax with 11 chaetigers (Fig. 2A, B). Thoracic segments biannulated, with shallow intra- and intersegmental grooves (Fig. 2A, B). Anterior five thoracic segments slightly expanded (Fig. 2A, B). First chaetiger biramous, with three or four bi-limbated capillaries; chaetigers 2–5 with six to 14 capillaries per fascicle in both parapodia; chaetigers 6–11 with five to 12 long-shafted hooded hooks per fascicle (Fig. 2A, B, F); thoracic hooks with indistinct node on shaft and at least six teeth in three rows above the main fang (Fig. 2F). Notopodia located dorso-laterally, dorsally located in last few thoracic segments; neuropodia located in lateral positions (Fig. 2A, B). Lateral organs present between noto- and neuropodia of all thoracic chaetigers, nearer to notopodia in chaetigers 5–11; sometimes indistinct on first thoracic chaetigers (Fig. 2A). Genital pores present in intersegmental furrows of between chaetigers 7–8, 8–9, 9–10, and 10–11 (Fig. 2A).

Transition between thorax and abdomen distinguished by changes in ultrastructure of chaetae and shape of segment (Fig. 2A, B); abdominal segments multi-annulated, gradually longer posteriorly, with short-shafted hooded hooks in posterior parapodial lobes; thoracic chaetigers usually bi-annulated, wider than long, with long-shafted hooded hooks in center of segment (Fig. 2A, B).

Abdominal parapodial lobes located in posterior end of each segment, well separated from each other, and gradually developed posteriorly (Fig. 2A–D). Abdominal notopodia separated, mid-dorsal on anterior few segments, becoming dorsolateral in following abdominal region, with six to eight hooded hooks per fascicle, having dorso-posteriorly protruded and hemispheric lobes from chaetiger 90 to end of body (Figs 2A–D, 5B). Abdominal neuropodia well separated, with 10–12 hooded hooks per fascicle, having slightly protruded lobes in posterior abdomen; neuropodial lobes less developed than notopodial lobes (Figs 2C, D, 5B).

Hooded hooks with main fang extending slightly beyond hoods. Abdominal hooks with distinct node on shaft and four rows of small teeth above main fang; three teeth in basal row, three in second and third row, and four to six in superior row (Figs 2E, G, 5A). Pygidium with digitate anal cirrus (Figs 2D, 5B).

###### Methyl green staining pattern.

Prostomium, peristomium and thoracic chaetigers 1–2 not stained (Fig. 6A). Thoracic chaetigers 3–11 stained blue; chaetigers 3–8 stained dark blue; chaetigers 9–11 stained light blue; post-chaetal region of chaetiger 11 not stained (Fig. 6A). Abdominal region without any distinct staining pattern.

###### Etymology.

The species is named for its wide distribution in Namhae (=Korean name of southern sea of Korea).

###### Distribution.

Subtidal areas (15–54 m) near southern part of Korea (Fig. 1).

###### Ecology.

*Heteromastus namhaensis* was sampled from soft sediments in March of 2016 (10 ind./m^2^), April of 2018 (40 ind./m^2^), and June of 2018 (20 ind./m^2^). The most well-developed individual (having over 100 segments) was obtained in March and eggs in the coelom were 87–94 μm in diameter. Surface sediment of the station was mainly composed of sandy mud with fragmented shells. *Leiochrides yokjidoensis* Jeong, Wi & Suh, 2017 co-occurred in Jejudo of Korea (Jeong et al. 2017a; Fig. 1). The salinity range among sampling locations was about 31–32.5 PSU.

###### Remarks.

*Heteromastus namhaensis* resembles *H. filiformis* sensu Hutchings & Rainer, 1982 in the absence of distinct eyespots on prostomium, three teeth in basal row above the main fang of abdominal hooks, and the presence of posteriorly extended abdominal notopodial lobes (Table 2). However, they differ in the shape of notopodial lobes in posterior abdomen (hemispheric protrusion in *H. namhaensis* vs broadly-based and rounded lamellae in *H. filiformis* sensu Hutchings & Rainer, 1982), the different dental structure of abdominal hooks (Table 2). *Heteromastus namhaensis* is also easily distinguished from Korean former record of *H. filiformis* (Choi and Yoon 2016) by the presence of hemispheric abdominal parapodial lobes and the absence of eyespots in *H. namhaensis*. In particular, the hemispheric notopodial lobe of *H. namhaensis* is a unique feature in the genus.

**Table 2.** Morphological comparison between *Heteromastus* species of this study and their closely related species. A: absent; P: present; Ch: chaetiger.

**Species**	**Eyespots**	**Dental structure of abdominal hooks**	**Notopodial lobes in posterior abdomen**	**Methyl green staining pattern**	**Habitat (locality)**
*H. namhaensis* sp. nov.	A	4 rows (3/3/3/4–6)	Hemispheric notopodial lobes dorso-posteriorly extended	Ch 3–11 blue, abdomen not stained	Subtidal, 36 m, sandy mud with shell fragments (Korea)
*H. gusipoensis* sp. nov.	A	4 rows (3/3/4/2)	Not extended	Ch 3–10 with blue speckles, median part of each segment stained densely	Intertidal, 0–1 m, sandy mud (Korea)
*H. koreanus* sp. nov.	P	3 rows (2/3/4)	Rounded notopodial lobes posteriorly extended	Ch 6–11 green, Ch 11 dark, abdomen not stained	Intertidal, estuarine, 0–1 m, sandy mud (Korea)
*H. filiformis* sensu Hutchings & Rainer, 1982	A	3 rows (3–4/4–5/4–6)	Broadly-based and rounded notopodial lobes posteriorly extended	Unknown	Intertidal (Mediterranean)
*H. filiformis* sensu Choi & Yoon, 2016	P	3–4 teeth in 3 rows	Rounded notopodial lobes posteriorly extended	Ch 1 & Ch 3–11	Intertidal (Korea)
*H. tohbaiensis* Yabe & Mawatari, 1998	P	Variable (>11)	Not extended	Unknown	Lake, low salinity, fine mud (Japan)

##### Heteromastus gusipoensis sp. nov.

Taxon classificationAnimaliaAnnelidaCapitellidae

F1F81C8F06275EBB8634327E44E29C2A

http://zoobank.org/0E904D4E-8DED-483D-A503-C60AFCB1671F

**Figure 3.**
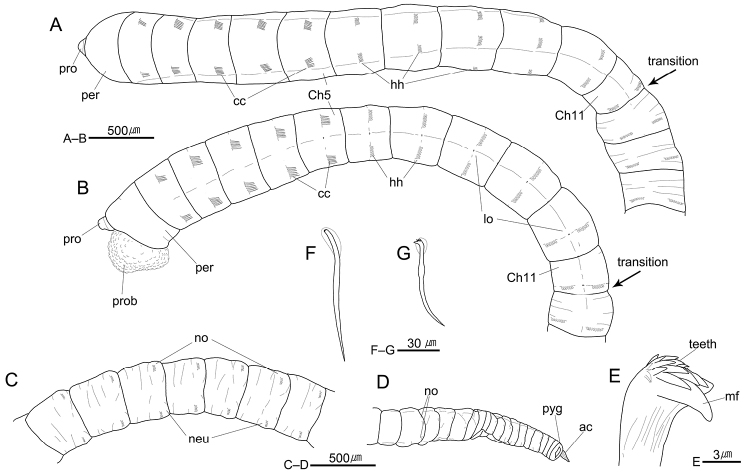
*Heteromastus gusipoensis* sp. nov. **A** anterior end, dorsal view (holotype, NA00155561) **B** same, lateral view **C** posterior abdominal segments, left lateral view (holotype, NA00155561) **D** posterior end, dorsal view (holotype, NA00155561) **E** abdominal short-shafted hook, frontal view **F** thoracic long-shafted hook, lateral view **G** abdominal short-shafted hook, lateral view. Abbreviations: ac, anal cirrus; cc, capillary chaetae; Ch, chaetiger; gp, genital pore; hh, hooded hooks; lo, lateral organ; mf, main fang; neu, neuropod; no, notopod; per, peristomium; pro, prostomium; prob, proboscis; pyg, pygidium.

Figures 3A–G , 5C, D , 6B

###### Material examined.

**Holotype**: MABIKNA00155561, sex uncertain, Yeonggwang, 35°25.819'N, 126°25.482'E, intertidal, tidal mud-flat, 1 m depth, November 2017, coll. Man-Ki Jeong. **Paratypes** (2 specimens): MABIK NA00155562 and NA00155563, same information as holotype.

###### Additional material examined.

MABIK NA00155564, sex uncertain, Yeonggwang, 35°25.819'N, 126°25.482'E, intertidal, tidal mud-flat, 1 m depth, May 2015, coll. Man-Ki Jeong. Additional 16 specimens from type locality on SEM stub.

###### Diagnosis.

Abdominal hooks with four rows of teeth; three teeth in basal row, three in second row, four in third row, and two in superior row. Genital pores present in intersegmental furrows of between chaetigers 5–6, 6–7, 7–8, 8–9, 9–10, and 10–11. Posteriorly extended parapodial lobes absent on abdominal segments.

###### Description.

Holotype entire, about 26 mm long, 0.5 mm wide for 120 chaetigers. Paratypes range from 19–24 mm in length, 0.4–0.5 mm width for 75–110 chaetigers. Body thread-like, rounded dorsally, flattened ventrally, widest in anterior thoracic chaetigers, and tapering from abdomen to pygidium. Color yellowish white in alcohol.

Prostomium short, conical, with short and blunt palpode; nuchal organs not seen, eyespots absent (Fig. 3A, B). Everted proboscis with small hemispheric papillae (Fig. 3B). Peristomium weakly bi-annulated and subequal in length with chaetiger 1 (Fig. 3A, B).

Thorax with 11 chaetigers (Fig. 3A, B). Thoracic segments biannulated, with shallow intra- and intersegmental grooves (Fig. 3A, B). First chaetiger biramous, with three or four bi-limbated capillaries; chaetigers 2–5 with six or seven capillaries per fascicle in both parapodia; chaetigers 6–11 with six or seven long-shafted hooded hooks per fascicle (Fig. 3A, B, F); thoracic hooks with indistinct node on shaft and at least 10 small teeth in three rows above the main fang (Fig. 3F). Notopodia located in dorso-laterally, dorsally located in last few thoracic segments; neuropodia located in lateral positions (Fig. 3A, B). Lateral organs present between both parapodia of all thoracic chaetigers, nearer to notopodia in chaetigers three to 11 (Fig. 3B). Genital pores present in intersegmental furrows between chaetigers 5–6, 6–7, 7–8, 8–9, 9–10, and 10–11; sometimes indistinct between chaetigers 5–6 (Fig. 3B).

Transition between thorax and abdomen distinguished by changes in chaetation and shape of segment (Fig. 3A, B); abdominal segments multi-annulated, with short-shafted hooded hooks in posterior part of segment; thoracic chaetigers usually bi-annulated, with long-shafted hooded hooks in center of segment; last thoracic chaetiger usually shorter than first abdominal chaetiger (Fig. 3A, B).

Abdominal parapodial lobes well separated from each other, located in posterior end of each segment (Fig. 3A–C). Abdominal notopodia separated, mid-dorsal on anterior few segments, becoming dorsolateral in following abdominal region, with five or six hooded hooks per fascicle, not protruded in anterior abdominal region, and very weakly protruded above epidermis in mid-posterior abdomen; not extended over further segment (Figs 3A–D, 5D). Abdominal neuropodia separated, not protruded, with six to eight hooded hooks per fascicle; neuropodial lobes less developed than notopodial lobes (Figs 3A–C, 5D).

Hooded hooks with main fang extending slightly beyond hoods. Abdominal hooks with distinct node on shaft and four rows of small teeth above main fang; three teeth in basal row, three in second row, four in third row, and two in superior row (Figs 3E, G, 5C). Pygidium with digitate anal cirrus (Figs 3D, 5D).

###### Methyl green staining pattern.

Prostomium, peristomium and thoracic chaetigers 1–2 not stained (Fig. 6B). Thoracic chaetigers 3–10 stained blue; blue speckles restrictively present on the median part of each segment; blue speckles sparse in chaetigers 3–4 (Fig. 6B). Abdominal region without any distinct staining pattern; parapodial lobes of chaetigers 12–13 slightly stained in blue but rapidly fades.

###### Etymology.

The new species is named for its limited distribution in Gusipo, Korea.

###### Distribution.

Intertidal area (0–1 m) near Gusipo, Korea.

###### Ecology.

*Heteromastus gusipoensis* was sampled in May of 2015 (9 ind./m^2^) and November of 2017 (71 ind./m^2^). Most well-developed individuals (having over 120 segments) were obtained in November. Surface sediment of the collecting station was mainly composed of fine sand and silt. Unidentified nereidid polychaetes co-occurred in the same location. The salinity of the sampling location was about 32.

###### Remarks.

*Heteromastus gusipoensis* closely resembles *H. tohbaiensis* Yabe & Mawatari 1998 in the chaetal arrangement and the absence of developed parapodial lobes in posterior abdomen (Table 2). However, they differ in the presence of eyespots on prostomium and distinct node on the shaft of thoracic hooks in *H. tohbaiensis* (Table 2; Yabe and Mawatari 1998). Moreover, they occur in different habitats and geographical areas. *H. gusipoensis* only occurs in the marine intertidal zone (salinity ca 32) of southwestern Korea, whereas *H. tohbaiensis* is only reported from the lacustrine habitat of northern Japan (Yabe and Mawatari 1998). *Heteromastus gusipoensis* is readily distinguished from the Korean former record, *H. filiformis* sensu Choi & Yoon, 2016, by the absence of prostomial eyespots and expanded abdominal parapodial lobes in *H. gusipoensis*.

##### Heteromastus koreanus sp. nov.

Taxon classificationAnimaliaAnnelidaCapitellidae

F0A1402D37865C809ED18E80FBC2D1E2

http://zoobank.org/C70CE167-A93A-45B1-AD5F-45DD159511C7

**Figure 4.**
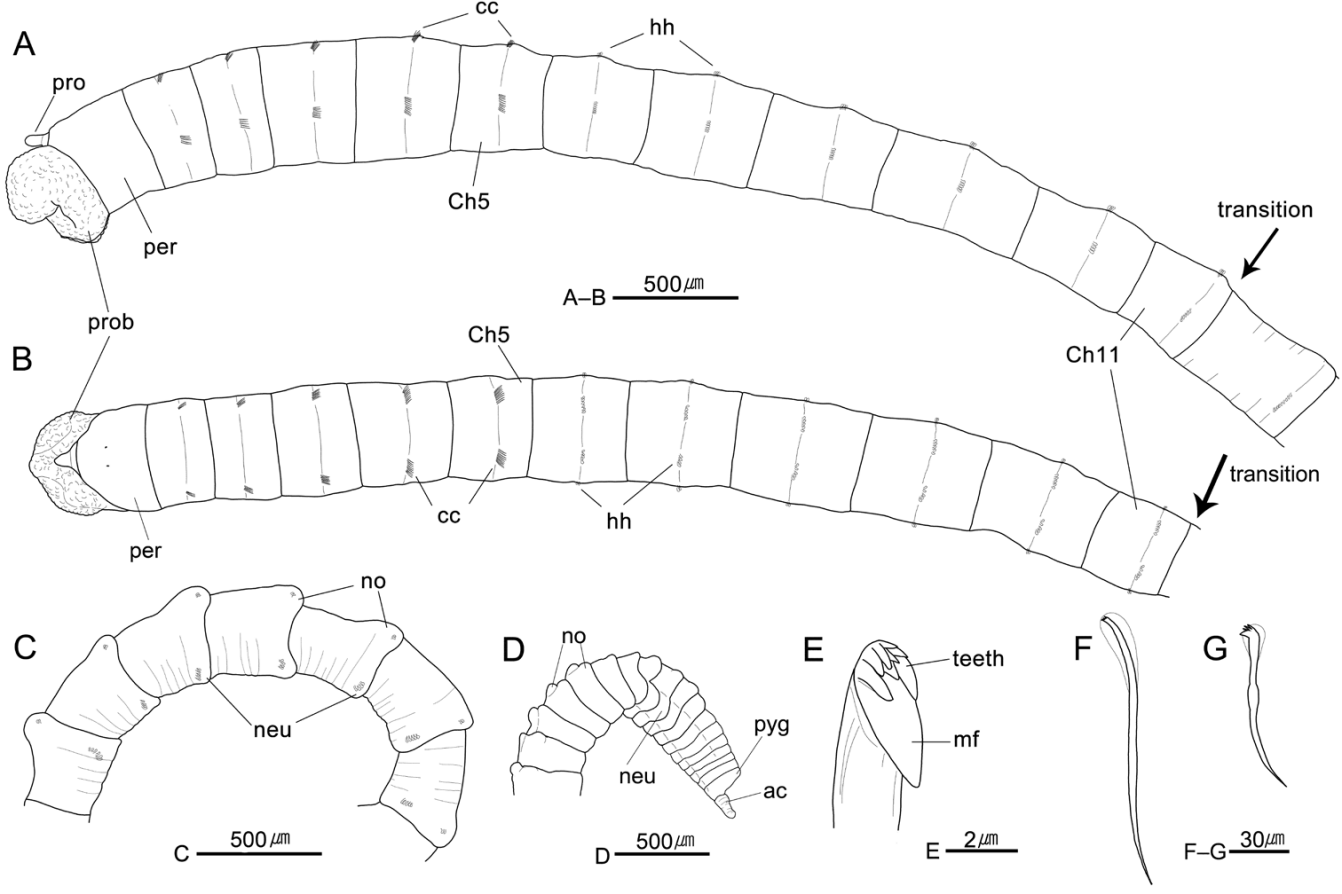
*Heteromastus koreanus* sp. nov. **A** anterior end, lateral view (holotype, NA00155565) **B** same, dorsal view **C** posterior abdominal segments, left lateral view (holotype, NA00155565) **D** posterior end, left lateral view (holotype, NA00155565) **E** abdominal short-shafted hook, frontal view **F** thoracic long-shafted hook, lateral view **G** abdominal short-shafted hook, lateral view. Abbreviations: ac, anal cirrus; cc, capillary chaetae; Ch, chaetiger; gp, genital pore; hh, hooded hooks; lo, lateral organ; mf, main fang; neu, neuropod; no, notopod; per, peristomium; pro, prostomium; prob, proboscis; pyg, pygidium.

**Figure 5.**
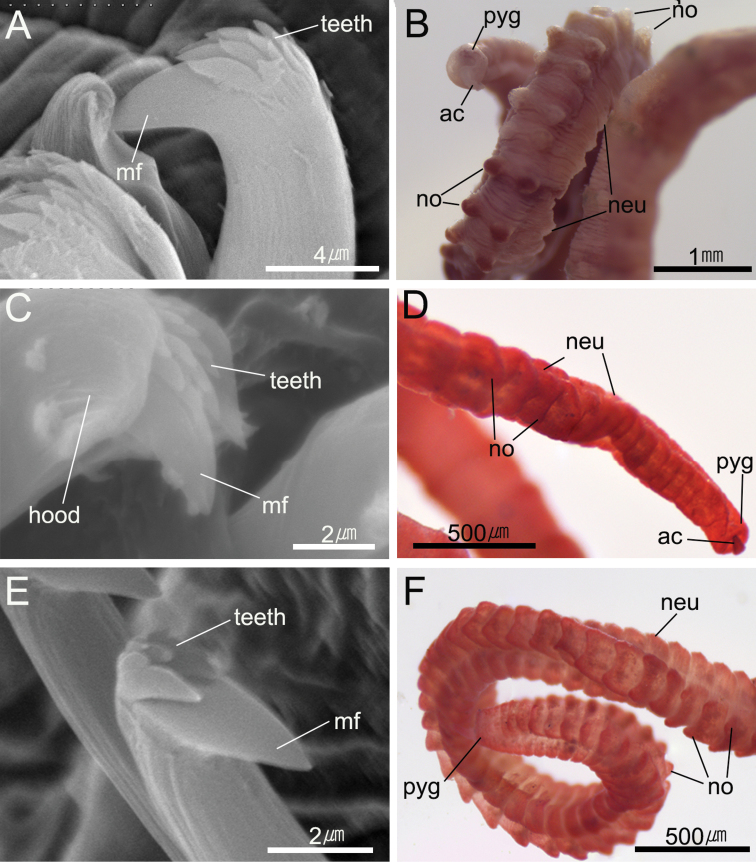
*Heteromastus namhaensis* sp. nov. **A** abdominal hooded hook in lateral view **B** posterior end in dorsal view (holotype, NA00155558). *Heteromastus gusipoensis* sp. nov. **C** abdominal hooded hook in frontal view **D** posterior end in dorsal view (holotype, NA00155561). *Heteromastus koreanus* sp. nov. **E** abdominal hooded hook in frontal view **F** posterior end in dorsal view (holotype, NA00155565). Abbreviations: ac, anal cirrus; mf, main fang; neu, neuropod; no, notopod; pyg, pygidium.

**Figure 6.**
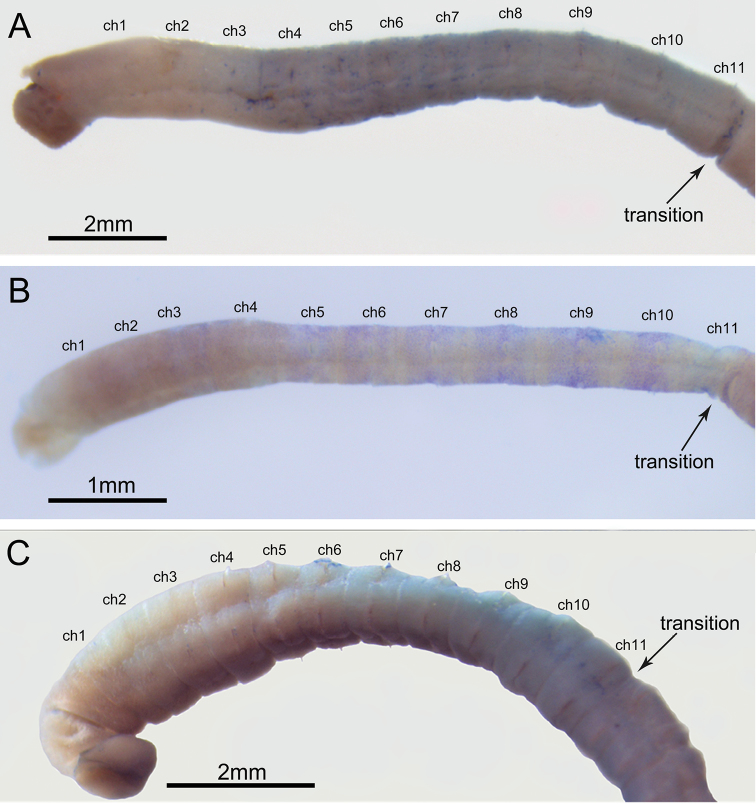
Methylene green staining patterns of Korean three new species **A** anterior end of *H. namhaensis* sp. nov., lateral view (paratype, NA00155560) **B** anterior end of *H. gusipoensis* sp. nov., lateral view (using additional specimens from type locality) **C** anterior end of *H. koreanus* sp. nov., lateral view (NA00065689).

Figures 4A–G , 5E, F , 6C

###### Material examined.

**Holotype**: MABIKNA00155565, sex uncertain, Muan, 35°6.270'N, 126°20.093'E, intertidal, tidal mud-flat, 1 m depth, November 2017, coll. Man-Ki Jeong. **Paratypes** (2 specimens): MABIKNA00155566, sex uncertain, Anheung, 36°40.740'N, 126°9.121'E, intertidal, muddy sand beach, 1 m depth, April 2014, coll. Man-Ki Jeong; MABIK NA00155567, sex uncertain, Gwangyang, 34°55.940'N, 127°36.252'E, intertidal, tidal mud-flat, 1 m depth, November 2017, coll. Man-Ki Jeong.

###### Additional material examined.

MABIKNA00155568, sex uncertain, Seochun, 36°0.95'N, 126°39.79'E, intertidal, tidal mud-flat, 1 m depth, May 2015, coll. Man-Ki Jeong. Additional seven specimens from type locality on SEM stub.

###### Diagnosis.

Abdominal hooks with three rows of teeth; two teeth in basal row, three in second row, and four in superior row. Genital pores present in intersegmental furrows between chaetigers 7–8, 8–9, 9–10, and 10–11. Posteriorly extended and rounded thin parapodial lobes present on posterior abdominal segments.

###### Description.

Holotype entire, about 28 mm long, 0.5 mm wide for 115 chaetigers. Paratypes range from 36–51 mm in length, 0.6 mm width for 89–95 chaetigers. Body thread-like, rounded dorsally, flattened ventrally, widest in anterior thoracic chaetigers, and tapering from abdomen to pygidium. Color whitish yellow in alcohol.

Prostomium conical, with slender and relatively long palpode; nuchal organs not seen, eyespots usually not observed in preserved specimen (Fig. 4A), sub-epidermal eyespots observed in few preserved specimens from Anheung of Korea (Fig. 4B). Everted proboscis with numerous small papillae (Fig. 4A, B). Peristomium uniannulated and slightly longer than chaetiger 1 (Fig. 4A, B).

Thorax with 11 chaetigers (Fig. 4A, B). Thoracic segments biannulated, with shallow intra- and intersegmental grooves (Fig. 2A, B). Anterior five thoracic segments slightly expanded (Fig. 4A). First chaetiger biramous, with three or four bi-limbated capillaries; chaetigers 2–5 with five to eight capillaries per fascicle in both noto- and neuropodia; chaetigers 6–11 with six to 10 long-shafted hooded hooks per fascicle (Fig. 4A, B, F); thoracic hooks with indistinct node on shaft and at least eight small teeth in three or four rows above the main fang (Fig. 4F).

Notopodia located in dorso-laterally, dorsally located in last few thoracic segments; neuropodia located in lateral positions (Fig. 4A, B). Lateral organs present between noto- and neuropodia of all thoracic chaetigers, nearer to notopodia in chaetigers 5–11 (Fig. 4A). Genital pores present in intersegmental furrows of between chaetigers 7–8, 8–9, 9–10, and 10–11 (Fig. 4A).

Transition between thorax and abdomen distinguished by changes in shape of chaetae and segment (Fig. 4A); anterior abdominal segments multi-annulated, gradually longer posteriorly, with short-shafted hooded hooks placed posteriorly in segment; posterior thoracic chaetigers bi- or tri-anullated, with long-shafted hooded hooks in central part of segment; last thoracic chaetiger smaller than first abdominal chaetiger (Fig. 4A).

Abdominal parapodial lobes located in posterior end of each segment, well separated from each other, and gradually developed posteriorly (Fig. 4C, D). Abdominal notopodia separated, middorsal on anterior few segments, becoming dorsolateral in following abdominal region, with 5 or 6 short-shafted hooded hooks per fascicle, having posteriorly extended and rounded thin lobes from chaetiger 70–80 to end of body; expanded notopodial lobes overlap dorso-anterior part of further segment (Figs 4C, D, 5F). Abdominal neuropodia well separated, with 10–12 short-shafted hooded hooks per fascicle, having slightly protruded lobes in posterior abdomen; neuropodial lobes less developed than notopodial lobes (Figs 4C, D, 5F).

Hooded hooks with main fang extending slightly beyond hoods. Abdominal hooks with distinct node on shaft and three rows of small teeth above main fang; two teeth in basal row, three in second row, and four in superior row (Figs 4E, G, 5E). Pygidium with digitate anal cirrus (Fig. 4D).

###### Methyl green staining pattern.

Prostomium, peristomium and thoracic chaetigers 1–5 not stained (Fig. 6C). Thoracic chaetigers 6–11 stained green (Fig. 6C). Abdominal region without distinct staining pattern; first two or three abdominal segments stained light green but rapidly fades; anal segment stained blue in well-developed specimens.

###### Etymology.

The new species is named for its wide distribution in coastal waters of Korea.

###### Distribution.

Intertidal areas (0–1 m) near Korea (Fig. 1).

###### Ecology.

*Heteromastus koreanus* was mainly sampled from Gwangyang in April of 2014 (35 ind./m^2^) and November of 2017 (470 ind./m^2^). Most well-developed individuals (having over 110 segments) were obtained from Muan and Gwangyang in November and coelomic eggs were 54–71 μm in diameter. Surface sediment of the collecting station was mainly composed of fine sand and silt. Unidentified cirratullid and nereidid polychaetes co-occurred in Gwangyang, Korea. The salinity range among sampling locations was about 15–33. Gwangyang is the only estuarine habitat. Other locations are situated in marine mud flats.

###### Remarks.

*Heteromastus koreanus* closely resembles former records of *H. filiformis* reported by Hutchings and Rainer (1982) and Choi and Yoon (2016) in the chaetal arrangement, the presence of posteriorly extended notopodial lobes in posterior abdomen, and the absence of the spine-like uncini and the distinct branchial structure (i.e. filamentous or digitiform) in posterior abdomen (Warren 1994; Blake 2000; Table 2). However, they differ in the dentition of abdominal short-shafted hooks (2/3/4 in *H. koreanus* vs 3–4/4–5/4–6 in *H. filiformis* sensu Hutchings & Rainer, 1982 vs three or four teeth in three rows in *H. filiformis* sensu Choi & Yoon, 2016), and the species-specific MGSP (Table 2). Additionally, *H. filiformis* occurs in the marine intertidal areas of Atlantic, Mediterranean, and America (Blake 2000) whereas *H. koreanus* of present study is collected mainly from the estuarine environment (salinity of 15–23) of Korea (Table 2). *Heteromastus koreanus* is also similar to *H. tohbaiensis* in the chaetal arrangement and presence of eyespots. However, they clearly differ in absence of distinct node on shaft of thoracic hooks and presence of expanded abdominal parapodial lobes in *H. koreanus* (Yabe 1998).

###### Molecular comparisons.

To verify the genetic divergence between examined specimens, partial sequences of mitochondrial (mtCOI) and nuclear (histone H3) genes were used. Intraspecific differences for mtCOI (MK032276–MK032284) and histone H3 (MK032285–MK032293) genes of each Korean species were very low (0–0.4%, Table 3). Based on mtCOI gene comparison, mean interspecific differences among these three new Korean species of the present study were distinct (16.0–18.9%, Table 3). All examined Korean *Heteromastus* species were well distinguished genetically from *H. filiformis* of China (13.3–19.6%, HZPLY183-12) and America (19.7–22.0%, MH235890). Based on histone H3 gene comparison, mean interspecific differences among the Korean *Heteromastus* species were 2.8–5.4% (Table 3). The known genetic difference for the mtCOI gene among capitellid species is 12.3–23.7% (Jeong et al. 2017b). In contrast, the published histone H3 gene difference between cryptic polychaetes is 2–9% (Glasby et al. 2013). Thus, genetic differences of these examined *Heteromastus* species (COI: 13.3–22.0%, H3: 2.8–5.4%) are significant at species level. Among all sequences of unidentified *Heteromastus* in Genbank database, sequences regarding two specimens from southern Japan (COI: LC208123–LC208124, H3: LC208100–LC208101) were genetically very close to *H. koreanus* of present study (COI gene difference: 2.1–3.3%, H3 gene difference: 0.9–1.3%). Among the described *Heteromastus* species from Japan, *H. tohbaiensis* resembles *H. koreanus* in the chaetal arrangement and presence of prostomial eyespots. However, they clearly differ in presence of distinct node on shaft of thoracic hooded hooks and absence of expanded abdominal parapodial lobes in *H. tohbaiensis* (Yabe 1998). Moreover, these two unidentified sequences (LC208123–LC208124) were originally reported from tidal mud flat and estuary near southern Japan, respectively (Tomioka et al. 2018). This distribution pattern is similar with those of *H. koreanus* (i.e. wide salinity range of 15–33) rather than *H. tohbaiensis*, which have been reported from lacustrine habitat of northern Japan. Despite the lack of morphological information regarding these Japanese specimens, the high similarity in genetic feature and inhabiting environment confirms the additional occurrence of *H. koreanus* in southern Japan.

**Table 3.** Mean genetic distances between examined *Heteromastus* species based on K2P distance. Bold numbers represent the mean intraspecific genetic distance of each species.

**mtCOI**	**1**	**2**	**3**	**4**	**5**
1. *H. namhaensis* sp. nov.(Korea)	**0.003**				
2. *H. gusipoensis* sp. nov. (Korea)	0.184	**0.001**			
3. *H. koreanus* sp. nov. (Korea)	0.189	0.160	**0.004**		
4. *H. filiformis* (China)	0.133	0.196	0.182	–	
5. *H. filiformis* (USA)	0.218	0.220	0.197	0.194	–
**histone H3**	**1**	**2**	**3**		
1. *H. namhaensis* sp. nov. (Korea)	**0.002**				
2. *H. gusipoensis* sp. nov. (Korea)	0.054	**0.000**			
3. *H. koreanus* sp. nov. (Korea)	0.048	0.028	**0.000**		

### Key to species of *Heteromastus*

**Table:** 

1	Thorax with 11 chaetigers; first chaetiger biramous; capillary chaetae only present on chaetigers 1–6	***H. giganteus* Zach, 1933**
–	Thorax with 11 chaetigers; first chaetiger biramous; capillary chaetae only present on chaetigers 1–5	**2**
2	Thoracic hooded hooks with distinct node on shaft	***H. tohbaienesis* Yabe & Mawatari, 1998**
–	Thoracic hooded hooks without distinct node on shaft	**3**
3	Abdominal hooks with node located posterior to middle of shaft	***H. similis* Southern, 1921**
–	Abdominal hooks with node located anterior to middle of shaft	**4**
4	Posterior abdominal segment with conspicuously projecting uncinial spines	***H. caudatus* (Hartman, 1976)**
–	Posterior abdominal segment without conspicuously projecting uncinial spines	**5**
5	Posterior abdomen with multiple filamentous branchiae	***H. filobranchus* Berkeley & Berkeley, 1932**
–	Posterior abdomen without multiple filamentous branchiae	**6**
6	Posterior abdomen with hemispheric and dorsally protruded notopodial lobes	***H. namhaensis* sp. nov.**
–	Posterior abdomen with thin notopodial lobes	**7**
7	Notopodial lobes on posterior abdomen not extended over following segment	***H. gusipoensis* sp. nov.**
–	Notopodial lobes on posterior abdomen overlap dorso-anterior part of following segment	**8**
8	Abdominal hooded hooks with at least 9 teeth above main fang; 2 distinct teeth in basal row	***H. koreanus* sp. nov.**
–	Abdominal hooded hooks with at least 11–15 teeth above main fang; 3 or 4 distinct teeth in basal row	***H. filiformis* sensu Hutchings & Rainer, 1982**
9	Hooded hooks with 7–8 teeth above main fang; 3 or 4 distinct teeth in basal row	***H. hutchingsae* Green, 2002**

## Supplementary Material

XML Treatment for Heteromastus

XML Treatment for Heteromastus namhaensis

XML Treatment for Heteromastus gusipoensis

XML Treatment for Heteromastus koreanus
